# Modulation, Plasticity and Pathophysiology of the Parallel Fiber-Purkinje Cell Synapse

**DOI:** 10.3389/fnsyn.2016.00035

**Published:** 2016-11-03

**Authors:** Eriola Hoxha, Filippo Tempia, Pellegrino Lippiello, Maria Concetta Miniaci

**Affiliations:** ^1^Neuroscience Institute Cavalieri Ottolenghi (NICO) and Department of Neuroscience, University of TorinoTorino, Italy; ^2^Department of Pharmacy, University of Naples Federico IINaples, Italy

**Keywords:** Purkinje cell, parallel fiber, synaptic plasticity, synaptic modulation, ataxia, AMPA receptor, mGlu_1_ receptor

## Abstract

The parallel fiber-Purkinje cell (PF-PC) synapse represents the point of maximal signal divergence in the cerebellar cortex with an estimated number of about 60 billion synaptic contacts in the rat and 100,000 billions in humans. At the same time, the Purkinje cell dendritic tree is a site of remarkable convergence of more than 100,000 parallel fiber synapses. Parallel fiber activity generates fast postsynaptic currents via α-amino-3-hydroxy-5-methyl-4-isoxazolepropionic acid (AMPA) receptors, and slower signals, mediated by mGlu_1_ receptors, resulting in Purkinje cell depolarization accompanied by sharp calcium elevation within dendritic regions. Long-term depression (LTD) and long-term potentiation (LTP) have been widely described for the PF-PC synapse and have been proposed as mechanisms for motor learning. The mechanisms of induction for LTP and LTD involve different signaling mechanisms within the presynaptic terminal and/or at the postsynaptic site, promoting enduring modification in the neurotransmitter release and change in responsiveness to the neurotransmitter. The PF-PC synapse is finely modulated by several neurotransmitters, including serotonin, noradrenaline and acetylcholine. The ability of these neuromodulators to gate LTP and LTD at the PF-PC synapse could, at least in part, explain their effect on cerebellar-dependent learning and memory paradigms. Overall, these findings have important implications for understanding the cerebellar involvement in a series of pathological conditions, ranging from ataxia to autism. For example, PF-PC synapse dysfunctions have been identified in several murine models of spino-cerebellar ataxia (SCA) types 1, 3, 5 and 27. In some cases, the defect is specific for the AMPA receptor signaling (SCA27), while in others the mGlu_1_ pathway is affected (SCA1, 3, 5). Interestingly, the PF-PC synapse has been shown to be hyper-functional in a mutant mouse model of autism spectrum disorder, with a selective deletion of *Pten* in Purkinje cells. However, the full range of methodological approaches, that allowed the discovery of the physiological principles of PF-PC synapse function, has not yet been completely exploited to investigate the pathophysiological mechanisms of diseases involving the cerebellum. We, therefore, propose to extend the spectrum of experimental investigations to tackle this problem.

## Introduction

While the physiological mechanisms of the parallel fiber-Purkinje cell (PF-PC) synapse are known in detail and its plasticity has been investigated in depth, the role, mechanisms and consequences of cerebellar neuromodulation are not completely known. Moreover, the pathophysiology of diseases caused by cerebellar dysfunction are only starting to be investigated, and the full spectrum of physiological methodologies has not yet been applied to these studies. This review is aimed at integrating the current knowledge about PF-PC synapse physiology, including its modulation, to help researchers to face the problem of the mechanisms responsible for cerebellum-dependent diseases including ataxia. In addition, we propose novel suggestions about new lines of research aimed at assessing the mechanisms of cerebellar involvement in autism.

## Physiological Properties of the Parallel Fiber-Purkinje Cell Synapse

The PF-PC synapse is the site of the greatest signal divergence in the cerebellar system. The afferent mossy fibers convey to the cerebellar cortex an enormous number of input signals representing almost every kind of information processed by the central nervous system. Each mossy fiber distributes signals to about 500 granule cells (Ito, 1984, 2006). Granule cells are the most abundant neuronal type in the central nervous system, with an estimated number of 92 × 10^6^ in the rat (Harvey and Napper, 1988) and 69 × 10^9^ neurons in the human cerebellum (Azevedo et al., 2009). Granule cell axons ascend toward the pial surface, and bifurcate in a T-shaped manner, giving rise to PFs. These fibers run in the transverse plane, along the major axis of a folium. In the rat cerebellum, PFs excite PC dendrites along an extension of about 3 mm (Arata and Ito, 2004; Ito, 2006). In the same animal species, it has been calculated that each PF forms about 300 *en passant* synapses with PCs (Palay and Chan-Palay, 1974; Ito, 2006). Thus, in the rat cerebellum, the signals originating from a single mossy fiber are distributed to about 150,000 PCs.

At the end of such a signal divergence, there is a massive convergence since each PC receives signals from more than 100,000 PFs (in the rat about 175,000; Napper and Harvey, 1988). For this reason, the extensive dendritic tree of PCs can be compared to a huge bidimensional matrix with more than 100,000 elements, one for each PF synaptic contact. The total number of PF-PC synaptic contacts in the whole cerebellum of rat corresponds to about 60 × 10^9^ (Huang et al., 2014). Concerning the human cerebellum, the number of PF-PC synaptic contacts can be estimated to be around 10^14^ (Azevedo et al., 2009; Huang et al., 2014). A series of theories concerning signal processing in the cerebellar cortex are based on the selection and fine tuning of this enormous variety of signals at the level of each single PF-PC contact (Marr, 1969; Albus, 1971; Schweighofer, 1998; D’Angelo et al., 2016).

In contrast to such richness of signals distributed by PFs, each PC receives inputs from a single climbing fiber (CF), which generates a powerful postsynaptic depolarization, giving rise to a brief train of action potentials, which together constitute the “complex spike”. The complex spike is associated with a large rise in intradendritic calcium (Ca^2+^) concentration (Konnerth et al., 1992; Miyakawa et al., 1992). Most theories of cerebellar function are based on the coincidence of PF-PC signals with the activity arriving via the CF (Marr, 1969; Albus, 1971; Ito, 1982, 2001). The time window for dynamic and plastic phenomena is further shaped by local inhibitory interneurons (Scelfo et al., 2008; Ramakrishnan et al., 2016). Thus, the modulation of PF-PC synapses and their short and long-term plasticity, together with PC intrinsic plasticity, are the crucial determinants of the way PCs process incoming signals to generate the output leaving the cerebellar cortex via their axons. In fact, PC axons are the sole efferent fibers from the cortex to the deep cerebellar nuclei (DCN), with the final effect of adding a contribution to a large variety of signals traveling in the central nervous system, including motor commands, cognitive, emotional, visceral and sensory information (for review see D’Angelo and Casali, 2013).

### The Parallel Fiber-AMPA-EPSC

The principal signal generated by glutamate released by PFs is a brief depolarization due to an excitatory post-synaptic current (EPSC) though postsynaptic glutamate receptors of the α-amino-3-hydroxy-5-methyl-4-isoxazolepropionic acid (AMPA) type, formed by subunits GluA1–4 (Tempia et al., 1996). The influx of Ca^2+^ through these receptors is negligible, as it has been measured to constitute about 0.6% of the total inward current. This means that, under physiological conditions, PC AMPA receptors are strongly selective for monovalent cations, such as sodium and potassium ions. The exclusion of Ca^2+^ ions from the selectivity filter is due to the presence of an arginine residue (R) in a critical position called Q/R site. The mechanism that allows PC AMPA receptors to have an R residue is peculiar, because all four genes of AMPA receptor subunits, GluA1, 2, 3 and 4, code for a glutamine (Q) residue (Hollmann et al., 1991; Hume et al., 1991). However, the GluA2 subunit undergoes an mRNA editing process, which converts a Q-coding triplet into and R-coding one (Sommer et al., 1991). In cerebellar PCs the efficiency of the editing process is almost 100%, as can be inferred by the negligible Ca^2+^ permeability of AMPA receptors. In addition to a complete editing of the GluA2 mRNA, a strong expression of this subunit is also necessary, so that virtually all AMPA receptors, which are tetramers, contain at least one R in the selectivity filter, which is sufficient to exclude Ca^2+^. This is the case in PCs.

It is interesting to note that the majority of PF-PC synapses have been shown to be silent but this has a series of advantages (Ekerot and Jörntell, 2001; Isope and Barbour, 2002). First, simulation studies using a perceptron neural network model have shown that the presence of 50% silent PF-PC synapses optimizes the information storage in terms of reliability and capacity (Brunel et al., 2004). Second, even if most PF-PC synapses are silent, the remaining synapses show a high release probability with only a few failures (Isope and Barbour, 2002). Third, PFs have a fast vesicle replenishment rate (Valera et al., 2012), which allows the active granule cells to transmit signals to PC with high efficiency and reliability (Isope and Barbour, 2002; Valera et al., 2012). These functional features endow the PF-PC synapse to sustain very high firing frequencies, up to 1 kHz, which are generated in bursts of activity during physiological stimulation *in vivo* (Eccles et al., 1966; Chadderton et al., 2004; van Beugen et al., 2013). In such a way PF-PC synapses can guarantee a high fidelity transmission even at high frequencies (Isope and Barbour, 2002; Valera et al., 2012), but also assure a linear transfer function for frequencies up to 300 Hz (van Beugen et al., 2013).

### The Parallel Fiber-mGlu_1_-EPSC

In addition to AMPA receptors located in front of the presynaptic active zone from which glutamate is released, PC dendritic spines innervated by PFs also express mGlu_1_ G-protein coupled receptors, localized perisynaptically (Nusser et al., 1994). As a consequence of their location, more distant from the release site, and because of the high efficiency of glutamate reuptake mechanisms (Takahashi et al., 1995), isolated action potentials are not effective for the activation of mGlu_1_ receptors, but a brief train of PF action potentials is required, so that glutamate concentration can build up in the synaptic cleft and diffuse to the perisynaptic zone. The activation of mGlu_1_ receptors generates a localized increase of Ca^2+^ in a portion of the dendritic tree (Finch and Augustine, 1998; Takechi et al., 1998; Tempia et al., 2001), accompanied by an excitatory post-synaptic potential (Batchelor and Garthwaite, 1993; Batchelor et al., 1994).

The mechanism responsible for the mGlu_1_-dependent intradendritic Ca^2+^ signal is the canonical transduction pathway. The resulting Ca^2+^ signal is independent of the membrane potential because it is generated by the release of Ca^2+^ from the endoplasmic reticulum (Finch and Augustine, 1998; Takechi et al., 1998; Tempia et al., 2001). The mGlu_1_ receptor activates a G_q_ protein, which activates phospholipase C (PLC), starting the production of diacylglycerol (DAG) and inositol 1,4, 5-trisphosphate (IP_3_). IP_3_ opens Ca^2+^ release channels (IP_3_ receptors: IP_3_Rs) in the endoplasmic reticulum. The coincident activation of PF leading to Ca^2+^ release, and CF, causing Ca^2+^ entry through voltage-gated channels, is considered a critical signal to trigger synaptic plasticity (Tempia and Konnerth, 1994).

The excitatory post-synaptic potential evoked by the activation of mGlu_1_ receptors (Batchelor and Garthwaite, 1993; Batchelor et al., 1994) is due to an inward current (PF-mGlu_1_-EPSC; Tempia et al., 1998). The PF-mGlu_1_-EPSC has an amplitude and a duration, which encode the frequency and the number of PF action potentials (Tempia et al., 1998). The latency of the PF-mGlu_1_-EPSC is in the subsecond range, relatively short for a G protein-mediated process, with the smallest value of 0.14 s (Tempia et al., 1998). The transduction pathway leading to the PF-mGlu_1_-EPSC is an unconventional one. PCs selectively express G proteins of the G_q_ subclass (Tanaka et al., 2000), and more precisely G_q_ and G_alpha11_ (Tanaka et al., 2000). Among these two proteins, only G_q_ is required for the generation of PF-mGlu_1_-EPSC (Hartmann et al., 2004); but its downstream pathway leading to the activation of PLC, IP_3_ or protein kinase C (PKC) is not involved (Tempia et al., 1998; Canepari et al., 2001). The ionic mechanism of the PF-mGlu_1_-EPSC is associated with a relevant sodium influx, which has been studied by optical methods (Knöpfel et al., 2000). As a result of several pioneering studies, hyperpolarization-activated cation channels (Canepari et al., 2001), purinergic receptors (Canepari et al., 2001), Na^+^/Ca^2+^-exchangers (Hirono et al., 1998) and voltage-gated Ca^2+^ channels (Tempia et al., 2001) have been excluded as molecular candidates responsible for the generation of the mGlu_1_-induced inward current in PCs. A few years later, it was shown that the mGlu_1_-induced current is in part mediated by a member of the class of canonical transient receptor potential (TRPC) channels (Kim et al., 2003), subsequently identified as TRPC3 (Hartmann et al., 2008). Recently, it has been proposed that activation of delta-2 glutamate (GluD2) receptors can also contribute to the PF-mGlu_1_-EPSC (Ady et al., 2014). GluD2 is an ion channel selectively permeable to the monovalent cations, unlike the TRPC3 channel, which has a non-selective permeability to Na^+^ and Ca^2+^, GluD2 is not gated by glutamate, so that for a long time it had been considered an orphan receptor, belonging to the glutamate receptors family merely on the basis of sequence similarity with the other members of this group. Since the mechanism of GluD2 gating upon glutamate binding to mGlu_1_ receptors is not known, further studies are necessary to confirm this finding.

Although the PF-mGlu_1_-EPSC does not require release of Ca^2+^ from internal stores of the endoplasmic reticulum (Tempia et al., 2001), it is strongly modulated by extracellular (Tempia et al., 1998, 2001; Tabata et al., 2002) and by intradendritic Ca^2+^ (Batchelor and Garthwaite, 1997; Tempia et al., 1997). Because of its sensitivity to intracellular Ca^2+^, PF-mGlu_1_-EPSC acts as coincidence detector. In fact, a train of PF action potentials, combined with an elevation of intradendritic Ca^2+^, causes a marked potentiation of the PF-mGlu_1_-EPSC (Batchelor and Garthwaite, 1997; Tempia et al., 1997; Dzubay and Otis, 2002). It should be pointed out that PCs possess several mechanisms for the control of intradendritic Ca^2+^. The first route of Ca^2+^ entry into PC dendrites is constituted by voltage-dependent Ca^2+^ channels. Thus, all mechanisms that depolarize PC dendrites enough to reach the threshold for Ca^2+^ channel gating can generate intradendritic Ca^2+^ signals. In fact, a strong activation of PFs can evoke a localized dendritic Ca^2+^ elevation (Eilers et al., 1995; Rancz and Häusser, 2006). However, the largest Ca^2+^ signals of PC dendrites are caused by the massive depolarization due to the activation of the CF-PC synapse, but in this case, the signal is spread throughout the dendritic tree (Konnerth et al., 1992; Miyakawa et al., 1992). Moreover, the coincidence of PF and CF activity has an additive effect leading to even larger elevations of intradendritic Ca^2+^ localized to the dendritic spines receiving both signals (Wang et al., 2000).

## Plasticity of the Parallel Fiber-Purkinje Cell Synapse

Long-term potentiation (LTP) and long-term depression (LTD) of the PF-PC synapses refer to long-lasting increase or decrease, respectively, of synaptic transmission. In the last decade, many studies have revealed that both forms of plasticity can occur *in vivo* and likely mediate some forms of learning, although the relationship between any one form of synaptic plasticity and a particular type of memory is still under debate (D’Angelo et al., 2016). These studies have also uncovered a wide range of induction mechanisms of synaptic plasticity, which converge not only on the presynaptic terminal where an enduring modification in the neurotransmitter release process takes place but also on the postsynaptic change in responsiveness to the neurotransmitter. It is now clear that presynaptic forms of LTP/LTD can co-exist with classical forms of postsynaptic plasticity at the PF-PC synapses but their induction and expression mechanisms appear to be almost completely distinct (see below). Interestingly, synaptic plasticity at PF-PC synapse can also be associated with persistent changes in intrinsic excitability of the same neurons, due to use-dependent modulation of voltage-gated ion channels (Mapelli et al., 2015). The combination of changes in synaptic strength and in intrinsic excitation can optimize information transfer within the cerebellar network and may thus contribute to the formation of the memory trace.

### Long-Term Depression (LTD)

**Figure 1:**
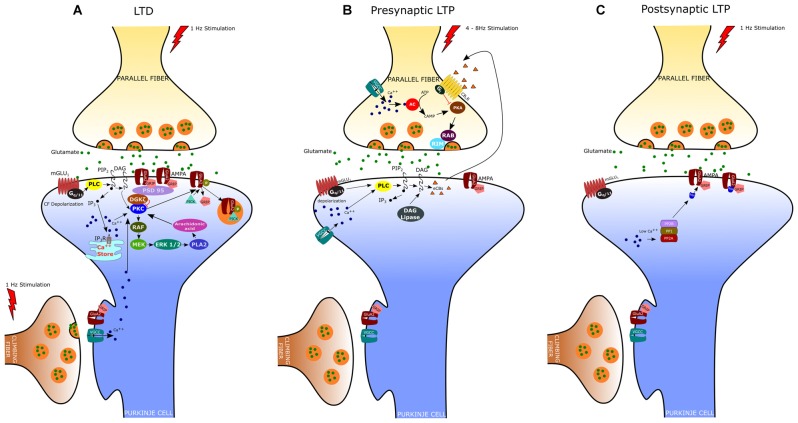
**Expression mechanisms of long-term depression (LTD), presynaptic Long-term potentiation (LTP) and postsynaptic LTP. (A)** LTD is induced by conjunctive stimulation of parallel and climbing fibers (CF) and requires postsynaptic Ca^2+^ elevation (see text). **(B)** Presynaptic LTP can be induced by brief PF stimulations at 4–8 Hz. A retrograde signaling mechanism mediated by cannabinoids regulates presynaptic LTP. The release of endogenous cannabinoids (eCBs) from the postsynaptic membrane is evoked by high-frequency bursts of parallel fiber activity and depends on the activation of postsynaptic mGlu_1_ receptors. eCBs act retrogradely onto presynaptic cannabinoid 1 receptors (CB_1_R), causing suppression of transmitter release. **(C)** Postsynaptic LTP can be induced by parallel fiber stimulation at 1 Hz and requires low levels of Ca^2+^ in the PC.

LTD at the PF-PC synapse is the best characterized form of synaptic plasticity in the cerebellum since it is believed to underlie several forms of motor learning, including the adaptation of the vestibulo-ocular reflex (VOR), associative eyeblink conditioning, and limb load adjustment (Ito, 1982, 1989; Krupa and Thompson, 1997). Indeed, an important feature of cerebellar LTD is the property of associativity: the long-lasting depression of PF responses results from the coincident activation of CF and PF inputs to a PC. LTD was originally described *in vivo* by Ito et al. (1982) in decerebrated rabbits. In this study, they demonstrated that stimulation of the vestibular nerve, which conveys mossy fibers to the flocculus, conjunctively with the stimulation of the inferior olive, the sole source of CFs, induced a long-lasting depression of the PC responses to vestibular nerve stimulation. Afterward, studies of *in vitro* preparations (i.e., cerebellar slices and cultures) have provided further insight into the cellular and molecular mechanisms of LTD (Ito, 2002). These studies indicate that PF-LTD requires the Ca^2+^ influx through P/Q voltage-gated channels, triggered by the CF-evoked depolarization, together with the release of glutamate by PFs, which acts upon both mGlu_1_ metabotropic receptors and AMPA receptors (Figure 1A). As reported above, stimulation of the mGlu_1_ receptor promotes, via G-protein (G_q/11_), DAG production and Ca^2+^ release from the endoplasmic reticulum (Aiba et al., 1994; Conquet et al., 1994; Matsumoto et al., 1996; Ichise et al., 2000; Hirono et al., 2001; Miyata et al., 2001; Hartmann et al., 2004; Kano et al., 2008). The PF-induced release of Ca^2+^ together with the Ca^2+^ entry evoked by CF stimulation determines a significant increase of Ca^2+^ concentration particularly at the level of spines and shafts of PC dendrites (Miyakawa et al., 1992; Eilers et al., 1995). DAG and Ca^2+^ act synergistically to activate PKC, which in turn activates a series of kinases, including Raf, MEK and ERK1/ERK2 (Tanaka and Augustine, 2008). Among the different PKC isoforms expressed in PCs, PKCα is critically required for LTD induction (Leitges et al., 2004). Indeed, cerebellar LTD is absent in PCs derived from PKCα null mice and is rescued by transfection with an expression plasmid encoding PKCα but not other PKC isoforms (Leitges et al., 2004). Activated PKC induces the phosphorylation of AMPA receptors at serin-880 of the GluA2 terminus, which results in the elimination of the receptor from the dendritic spines via clathrin-mediated endocytosis (Wang and Linden, 2000). The reduction in the number of postsynaptic AMPA receptors represents the key change for LTD expression since it is responsible for the reduced responsiveness to glutamate and therefore for the decreased transmission at the PF-PC synapse. It is now clear that the endocytic removal of postsynaptic AMPA receptors is a complex phenomenon regulated by the interaction of GluA2 not only with PKC but also with glutamate receptor-interacting proteins (GRIP1 and GRIP2/ABP) and the PDZ domain-containing protein PICK1 (protein interacting with C kinase 1). In particular, it has been demonstrated that GluA2 binds GRIP1 and GRIP2 in the dephospho-Ser-880 state; however, when GluA2 Ser-880 is phosphorylated by PKC, the affinity of GRIP1/2 for GluA2 is disrupted and this allows the binding of the receptor to PICK1 (Hirai, 2001). PICK1 is indeed considered a key regulator of AMPA receptor traffic since it is directly involved in the removal of AMPA receptors from the synaptic plasma membrane (Hanley, 2008). Recently, it has been found that the targeting of PKCα to the synapses is also regulated by a diacylglycerol kinase ζ (DGKζ), that can interact with PKCα and as well as with postsynaptic density protein 95 (PSD-95; Lee et al., 2015). DGKζ has the ability to metabolize DAG thus reducing the PKCα activity to the basal level. Following LTD induction, the activated PKCα phosphorylates and releases DGKζ so that PKCα can interact with PICK1 to enhance AMPA receptors internalization. By recruiting Raf kinase, PKC can also induce the sequential activation of MEK, ERK1/2 and phospholipase A_2_, resulting in the production of arachidonic acid and subsequent activation of PKC. The MAPK-PKC positive feedback loop is likely responsible for the sustained PKC activation and the maintenance of LTD (Yamamoto et al., 2012).

### Presynaptically Expressed LTP

In addition to LTD, PF-PC synapses can undergo both pre- and postsynaptically expressed types of LTP (Figures 1B,C). The presynaptically expressed LTP is induced by brief PF stimulations at the frequency of 4–8 Hz. The leading event is the increase of Ca^2+^ influx within PF terminals that activates a Ca^2+^/calmodulin-dependent adenylyl cyclase, resulting in enhanced presynaptic cAMP levels and protein kinase A (PKA) activation (Salin et al., 1996; Chen and Regehr, 1997; Storm et al., 1998; Jacoby et al., 2001). PKA then phosphorylates the vesicle-release related proteins, RIM1α and Rab3, thus increasing glutamate release from PFs (Kaeser et al., 2008). Although most of the presynaptic Ca^2+^ enters PFs through N-type and P/Q-type Ca^2+^ channels, PF-LTP can still occur when both of these channel types are blocked and the global Ca^2+^ levels are largely reduced by 50% (Myoga and Regehr, 2011). On the other hand, blocking R-type Ca^2+^ channels disrupts PF-LTP, despite these channels play a minor role in basal transmission and contribute modestly to overall Ca^2+^ entry. Presynaptic LTP appears to be finely regulated by endocannabinoids (eCB), which are released by PCs following PF stimulation (Maejima et al., 2001, 2005; Brown et al., 2003). Activation of CB_1_ receptors on PF terminals inhibits AC and PKA activity, thus preventing LTP induction. Interestingly, the coactivation of CF can enhance eCB retrograde inhibition of PF transmission thus promoting PF-LTD while suppressing PF-LTP (van Beugen et al., 2006).

### Postsynaptically Expressed LTP

The postsynaptically expressed LTP is evoked by PF stimulation at low frequency (typically 1 Hz) that triggers the GluA2-containing AMPA receptors insertion in the spine membrane. The GluA2 subunit insertion at synaptic sites involves N-ethylmaleimide-sensitive factor (NSF), an essential component of SNARE-mediated fusion machinery, which binds GluA2 and reduces the receptor internalization by PICK1 (Anggono and Huganir, 2012). In contrast, during LTD, the elevated postsynaptic [Ca^2+^] inhibits NSF–GluA2 interaction, thus promoting GluA2–PICK1 binding and synaptic removal of GluA2-containing AMPA receptors. Unlike LTD, the induction of postsynaptic LTP depends on lower Ca^2+^ transients and requires the activation of protein phosphatases PP1, PP2A and PP2B (Belmeguenai and Hansel, 2005). These phosphatases enhance GluA2 binding to GRIP, which in turn stabilizes the AMPA receptors to the membrane. Indeed, mutant mice, in which the PP2B (or calcineurin) was deleted only in cerebellar PCs, show deficit in postsynaptic PF-PC LTP and motor learning, whereas LTD induction was unaffected (Schonewille et al., 2010).

### Parallel Fiber-Purkinje Cell Plasticity in Motor Learning

The observation of concomitant impaired LTP and motor learning, with no alteration of LTD, challenged the common view that cerebellar LTD alone underlies motor learning. Indeed, cerebellar motor learning during eyeblink conditioning and adaptation of the VOR appears to be completely normal in mutants in which LTD induction was impaired by inactivation of AMPA receptor internalization (Schonewille et al., 2011). This suggests that learning may result from the interaction of several forms of synaptic plasticity and not just from classical LTD alone. Indeed, it is now clear that different forms of plasticity take place in the granular layer, in the molecular layer and DCN involving both excitatory and inhibitory synaptic transmission (Gao et al., 2012; D’Angelo et al., 2016). These forms of synaptic plasticity are believed to operate in conjunction with long-lasting modifications in neuronal excitability during memory formation in the cerebellum (Schreurs et al., 1998; Belmeguenai et al., 2010; Grasselli et al., 2016). Indeed, Purkinje cell recordings from cerebellar slices of rabbits that had acquired delay eyelid conditioning revealed an increased PC membrane excitability that lasted for 30 days after training.

But what is then the role of CFs? The principal hypothesis is that the complex spike discharge of PCs encodes motor error signals and serves as a teaching signal that drives motor adaptation. This means that LTD should be driven by error-related signals carried by CFs whereas LTP should occur when such error signals are absent, without CF supervision (Sakurai, 1987). A corollary of this hypothesis is that the CFs may regulate PC activity under certain conditions, such as when attention is enhanced or errors in motor execution become large, as suggested Kimpo et al. (2014). In their study, monkeys sat in a rotating chair (to provide vestibular stimuli), and were trained to track a visual object with their eyes. They found that when the visual stimulus was moved in the opposite direction from the vestibular stimulus so that a bigger reflexive eye movement was required to stabilize the image (VOR-increase training), the activation of the CFs in response to the error led to a change in the response of the PCs. However, when the visual stimulus moved together with the head and a smaller reflexive eye movement was needed (VOR-decrease training), the error signals carried by CFs did not alter the PC firing rate. Interestingly, the optogenetic activation of CFs, mimicking the visual error signals provided by retinal slip, was able to induce VOR-increase but not VOR-decrease learning. These results indicate that the CF activity and the plasticity of the PC responses are not so correlated during VOR-decrease training, as they are during VOR-increase learning. Thus, although both motor learning paradigms elicited error signals in the CFs, PCs could regulate the CF-triggered plasticity.

Moreover, there is growing evidence that long-term plastic changes can occur in the cerebellar cortical network in the absence of the intervention of CFs. For example, Ramakrishnan et al. (2016) have recently demonstrated that theta burst tactile stimulation (a burst of 80 air puffs delivered at 4 Hz) mimicking natural stimulation patterns can induce a long-lasting potentiation of PC discharge and long-lasting decrease of molecular layer interneurons firing in the absence of complex spike changes. Interestingly, there are also indications that memory of fear is accompanied by LTP at PF-PC synapses, which is consistent with the view that Hebbian learning occurs in the cerebellar cortex during fear conditioning (Sacchetti et al., 2004; Zhu et al., 2007). This fear conditioning-induced potentiation is postsynaptically expressed and displays some properties of LTP elicited *in vitro* by repetitive stimulation of PFs.

## Modulation of the Parallel Fiber-Purkinje Cell Synapse by Neurotransmitters

The cerebellar cortex receives projections from multiple neuromodulatory neurons releasing acetylcholine, norepinephrine, and serotonin (Schweighofer et al., 2004). The cell bodies of these neuromodulatory neurons are mainly grouped in specific nuclei in the brainstem, and through their widespread projections, they distribute to all parts of the cerebellar circuitry, i.e., cerebellar cortex and DCN. Because of this widespread projection, the neuromodulatory system can have profound effects on excitability and synaptic plasticity of target neurons (Ito and Schuman, 2008). Indeed, electrophysiological and behavioral studies have demonstrated that neuromodulation is implicated in cerebellar processing as well as cerebellar-dependent learning and memory.

### Serotonin

The cerebellum receives a dense innervation by serotonergic fibers, which represent the third largest population of afferent fibers extending into this brain region, after mossy and CFs. The serotonin inputs to the cerebellum originate mainly in the medullary and pontine reticular formation and distribute to both cerebellar cortex and nuclei (Chan-Palay, 1975; Bishop and Ho, 1985). Anatomical and pharmacological studies indicate that the cerebellar cortex expresses multiple subtypes of 5-hydroxytriptamine receptors (5-HTRs). In particular, there is clear evidence that PCs express the 5-HT_1_, 5-HT_2_, 5-HT_5_ and 5-HT_7_ subtypes whereas 5-HT_3_Rs and 5-HT_6_Rs are localized on granule cells (Pazos and Palacios, 1985; Geurts et al., 2002; Li et al., 2004). Expression of 5-HT_5A_R has also been found on Golgi cells and molecular layer interneurons (Geurts et al., 2002; Oostland et al., 2014). Given the widespread distribution of serotonergic innervation and the richness of signals evoked by different 5-HTR subtypes, it is not surprising that 5-HT has the potential to modulate both excitatory and inhibitory synaptic signals throughout the cerebellar network. Indeed, it has been shown that activation of 5-HT_1_Rs determines a general suppression of cerebellar cortex activity by reducing the release of the excitatory transmitter glutamate from PFs to PCs (Maura et al., 1986) and by increasing inhibitory synaptic transmission onto PCs (Mitoma et al., 1994; Mitoma and Konishi, 1999).

**Figure 2:**
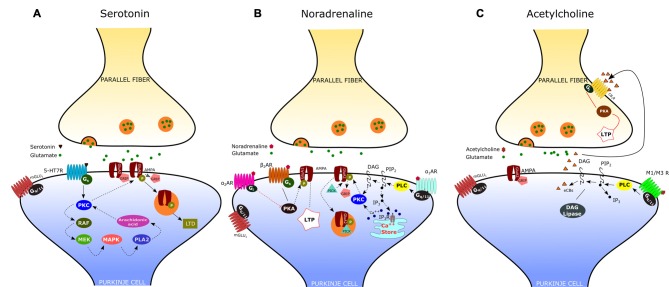
**Neuromodulation of the parallel fiber-Purkinje cell (PF-PC) synapse. (A)** Activation of the serotonin receptor 5-HT_7_R elicits the depression of the PF-PC synapse via a postsynaptic mechanism that involves the protein kinase C (PKC)-MAPK signaling pathway (see text). **(B)** α-adrenergic receptors (α_1_-ARs) exert an inhibitory effect on PF-PC synaptic transmission via G_q/11_ and phospholipase C (PLC). α_2_-ARs are known to inhibit adenylyl cyclase, via G_i_-proteins, leading to a decrease of cAMP concentration and protein kinase A (PKA). β-ARs activation facilitates the PF-PC synaptic transmission via the cAMP-PKA signaling pathway. **(C)** Acetylcholine induces a transient depression of PF-excitatory post-synaptic currents (EPSCs) via M_1_/M_3_ postsynaptic muscarinic receptors, which are coupled to G_q_ and linked to the cannabinoid signaling pathway.

By using patch-clamp recordings in cerebellar slices of adult mice, Lippiello et al. (2016) have recently demonstrated that 5-HT_7_Rs are critically implicated in synaptic plasticity of the PF-PC synapse, thus providing a novel cellular and molecular basis for the action of 5-HT at cerebellar level. In particular, they found that 5-HT_7_R activation by a selective agonist causes LTD of the PF-PC synapse via a postsynaptic mechanism that involves the PKC-MAPK signaling pathway (Figure 2A). As previously described, MAPK activation may trigger a positive feedback, via phospholipase A_2_, which is responsible for a sustained activation of PKC and consequent internalization of AMPA receptors. In addition, they showed that treatment with a 5-HT_7_R antagonist reduced the expression of postsynaptic PF-LTD, produced by pairing PF stimulation with PC depolarization; on the other hand, application of a 5-HT_7_R agonist impaired postsynaptic LTP induced by 1 Hz stimulation of PFs. These results suggest that 5-HT_7_R exerts a fine regulation of bidirectional synaptic plasticity by favoring the emergence of LTD vs. LTP at PF-PC synapses. This type of synaptic control may enable the serotonergic pathways to prevent the simultaneous occurrence of conflicting forms of plasticity at PF-PC, such as potentiation of synaptic transmission under conditions that promote postsynaptic LTD.

The involvement of serotonin 5-HT in motor learning has been observed in several cerebellar-dependent paradigms. For example, depletion of brain 5-HT has been shown to impair the horizontal VOR adaptation in rabbits (Miyashita and Watanabe, 1984). In addition, application of a 5-HT receptor (5-HTR) antagonist, ritanserin, to the cerebellar vermis before training, impaired the formation of long-term memory of conditioned freezing and extinction of the defensive component of the acoustic startle reaction (Storozheva and Proshin, 2011). Furthermore, chronic treatment with buspirone, a 5-HT1AR partial agonist, improves the motor coordination deficits in Lurcher mouse, a model of cerebellar neurodegeneration (Le Marec et al., 2001). Serotonergic modulation of the cerebellum has been also a subject of clinical interest since the discovery that long-term administration of L-5-hydroxytryptophan, a precursor of 5-HT, improves the dysfunctions associated with cerebellar disorders in patients with inherited or acquired ataxia (Trouillas et al., 1988, 1995). Furthermore, there is evidence that neurodevelopmental disorders such as autism and schizophrenia are associated with a change in 5-HTR expression in the cerebellum (Slater et al., 1998; Eastwood et al., 2001).

### Noradrenaline

The noradrenergic innervation of the cerebellum originates from the *locus coeruleus* and distributes to all parts of the cerebellar cortex and DCN (Olson and Fuxe, 1971; Abbott and Sotelo, 2000). Studies using lesions and pharmacological approaches combined with behavioral analysis have provided a direct evidence that the noradrenergic *locus coeruleus* system is implicated in a wide variety of brain processes, including attention, arousal, decision making and memory (Aston-Jones et al., 1999; Clayton et al., 2004). The demonstration that the cerebellar cortex receives a widespread projection from the *locus coeruleus* has led researchers to hypothesize that the noradrenergic system may also be involved in the modulation of cerebellar functions and motor learning. To test this hypothesis, van Neerven et al. (1990) examined the effect of the noradrenergic system on the adaptive changes of the VOR in rabbits. They found that the injection of a β-adrenergic agonist, isoproterenol, into the flocculus enhanced the adaptive potentiation of VOR particularly in darkness; whereas the application of a β-antagonist, sotalol, significantly reduced the adaptation of the VOR in the light and darkness. Further studies in rabbit and rat have demonstrated that blockade of β-adrenergic receptors (β-ARs) through systemic administration of propranolol impaired the acquisition of the eyeblink conditioning (Gould, 1998; Cartford et al., 2002). Abnormal levels of noradrenaline (norepinephrine: NE) have been noted in the cerebella of patients with olivocerebellar atrophy, Parkinson’s and Alzheimer’s diseases (Kish et al., 1984a,b; Shimohama et al., 1986).

*In situ* hybridization studies indicate that the cerebellar cortex expresses mRNA encoding all subtypes of α_1_-AR (α_1A_, α_1B_, α_1D_) and α_2_-AR (α_2A_, α_2B_, α_2C_; Schambra et al., 2005). These results are confirmed by immunostainings analyses showing a strong expression of α_1A_-AR as well as α_2A_- and α_2B_-AR in both molecular layer interneurons and PCs (Papay et al., 2004, 2006; Hirono et al., 2008). High-level expression of β_2_-AR has been also observed in PC bodies and molecular layer (Lippiello et al., 2015).

According to electrophysiological studies, iontophoretically applied NE or activation of the locus coeruleus induces depression of spontaneous discharges in PCs (Hoffer et al., 1971; Siggins et al., 1971). Such effect is associated with the potentiation of inhibitory GABAergic transmission at basket cell-PC synapses, mediated by presynaptic β_2_-ARs (Mitoma and Konishi, 1999; Saitow and Konishi, 2000). Interestingly, NE exerts also an inhibitory effect on the CF-PC synapse, acting presynaptically (on α_2_-ARs) to decrease glutamate release from CFs (Carey and Regehr, 2009). Concerning the noradrenergic effect on the PF-PC synapse, early reports have shown an inhibitory action of NA, mediated by α_2_-ARs, on the field potential evoked by PF stimulation (Mitoma and Konishi, 1999). Recent evidence indicates that noradrenaline functions as an endogenous ligand for both α_1_- and α_2_-ARs to produce synaptic depression between PFs and PCs, through a postsynaptic mechanism. (Figure 2B; Lippiello et al., 2015). This result is consistent with the immunochemical localization of both α_1_- and α_2_-ARs at the Purkinje cell dendrites (Papay et al., 2004, 2006; Hirono et al., 2008). The inhibitory effect α_1_-ARs on PF-PC synaptic transmission is likely related to the sequential activation of G_q/11_ and PLC which may induce DAG production and release of Ca^2+^ from intracellular stores. This may result in the activation of PKC and eventually phosphorylation of GluA2 and internalization of AMPA receptors (Herold et al., 2005). α_2_-ARs are known to inhibit adenylyl cyclase, via G_i_-proteins, leading to a decrease in cAMP concentration and PKA activity. A decrease in PKA activity determines the dephosphorylation of GluA1, which is considered a signal for internalization and synaptic depression (Yi et al., 2013). On the other hand, stimulation of the β-AR by isoproterenol determines a significant increase of PF-EPSCs (Lippiello et al., 2015). This short-term potentiation is postsynaptically expressed, requires PKA, and is mimicked by the β_2_-AR agonist clenbuterol. Interestingly, activation of β-ARs facilitates PF-PC synaptic plasticity by enhancing the ability of low-threshold stimuli to induce postsynaptic LTP. The mechanism by which β-ARs facilitate synaptic transmission may involve the cAMP-PKA signaling pathway. Indeed it has been demonstrated that PKA can phosphorylate GluA1 on S845 thus promoting AMPA receptor insertion into synapses (Joiner et al., 2010). Such bidirectional regulation of PF-PC synaptic transmission by NE with facilitation via β_2_-ARs and inhibition via α_1_-and α_2_-ARs may allow the noradrenergic inputs to finely regulate the signals arriving at PCs at particular arousal states or during learning.

### Acetylcholine

The presence of cholinergic innervation in the cerebellum has been demonstrated by earlier immunocytochemical studies using antibodies against choline acetyltransferase (ChAT). Such analysis combined to retrograde tracing has revealed a significant amount of ChAT-positive mossy fibers in the flocculo-nodular lobe, originating mainly from the vestibular nuclei, and a moderate number of ChAT-immunoreactive fibers in the DCN (Barmack et al., 1992; Jaarsma et al., 1997). Autoradiographic and immunohistochemical analysis have later provided evidence that the cerebellum expresses both muscarinic and nicotinic cholinergic receptors. Muscarinic receptors are mainly M_2_-type and are localized throughout the whole cerebellar cortex, particularly in the molecular layer of nodulus and ventral uvula, and in the cerebellar nuclei. On the other hand, the cerebellum expresses several subtypes of nicotinic acetylcholine receptors (nAChRs), with a predominance of the homomeric α_7_-nAChRs and the heteromeric nAChRs composed of α_3_ or α_4_ subunits in pairwise combination with either β_2_ or β_3_ subunits (Turner and Kellar, 2005). A specific nAChR expression has been observed in interneurons, granule cells and mossy fibers. These results are consistent with electrophysiological studies showing an increase of GABA release from Golgi cells and basket/stellate interneurons following activation of nAChRs (Rossi et al., 2003). Furthermore, it has been demonstrated that nicotine increases mossy fiber-granule cell synaptic transmission by acting on α_7_-nAChRs located at pre- and postsynaptic sites. On the other hand, Rinaldo and Hansel (2013) have recently demonstrated that muscarinic acetylcholine receptors (mAChRs) activation by a selective agonist induces a transient depression of PF-AMPA-EPSCs via a presynaptic effect mediated by M_3_ receptors. Interestingly, mAChR activation has also a suppressive effect on presynaptic LTP induction at PF–PC synapses via retrograde eCB signaling. According to their results, the eCB synthesis and release occurs at the postsynaptic site and is triggered by G_q_-coupled M_1_/M_3_ receptors expressed on PCs (Figure 2C). The effect of eCB is likely mediated by cannabinoid 1 receptors (CB_1_Rs), located in presynaptic terminals and consists in a reduced release of neurotransmitter through a G_i/o_-coupled pathway. Therefore, by activating multiple cholinergic receptor subtypes on different cellular targets, acetylcholine has the potential to modulate the cerebellar information processing within the cerebellar cortex.

Evidence for a role of cholinergic signaling in cerebellar motor learning has come from behavioral studies showing that the injections of muscarinic agonists into the vestibulocerebellum influenced the gains of optokinetic and vestibuloocular reflexes in the rabbit and the VOR gain in the decerebrate cat (Andre et al., 1992; Tan et al., 1993; van der Steen and Tan, 1997). The effect of nicotine has also been studied in human subjects scanned by functional magnetic resonance while performing an auditory-paced finger tapping task (Wylie et al., 2013). The task consisted in pressing a button with their right hand in response to an auditory cue at a constant rate of 1, 2 or 4 Hz. When compared to placebo treatment, subjects that received nicotine treatment showed an increased tapping rate, as a consequence of increased coordination, associated with an increased activity in the vermal area of the anterior cerebellum during the task.

nAChRs are also involved in developmental plasticity in the chick cerebellum and neurodevelopmental disorders such as autism (Kaneko et al., 1998). Indeed, developmental synaptogenic events are often accompanied by an increase in PC α_7_ nAChR immunoreactivity. On the other hand, an abnormal expression of nAChR α_4_ and α_7_ has been observed in the cerebellar cortex of autistic subjects compared to normal individuals (Lee et al., 2002).

## Alterations of the Parallel Fiber-Purkinje Cell Synapse in Neurologic and Psychiatric Diseases

### Spino-Cerebellar Ataxias

**Figure 3:**
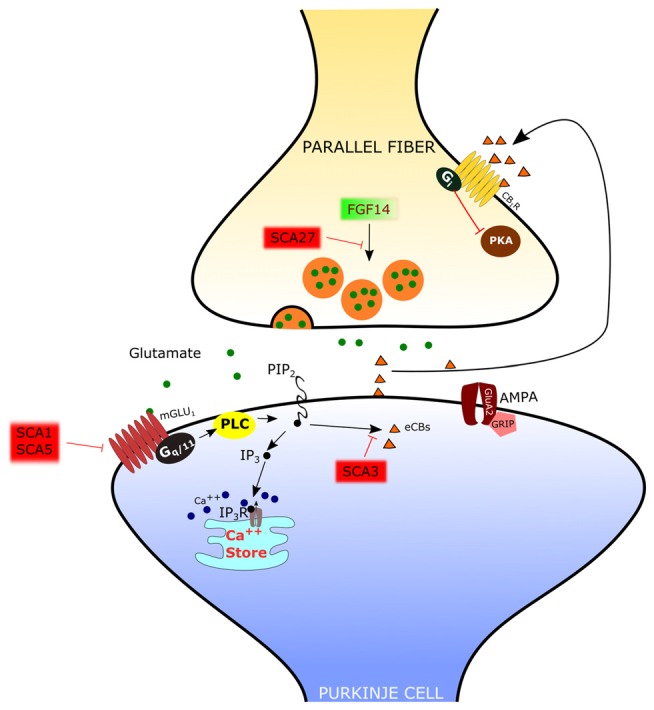
**Mechanisms of spino-cerebellar ataxias (SCA) affecting the PF-PC synapse.** In SCA1 and SCA5, the mGlu_1_ receptor is dysregulated. In SCA3 the endocannabinoid-dependent suppression of excitation evoked by PF stimulation is absent. Since this response requires mGlu_1_ activation, it has been hypothesized that signaling through this receptor is impaired. In SCA27 the release of glutamate by PFs is deficient.

The PF-PC synapse is involved in several disorders with altered cerebellar function. Regarding neurologic diseases, most studies have been focused on spino-cerebellar ataxias (SCAs), hereditary forms of cerebellar disorder with an autosomic dominant transmission pattern and with a variable involvement of extra-cerebellar structures. At present, the mutations responsible for more than 40 distinct types of SCAs have been described (Durr, 2010), but the cause of a large fraction of SCAs (20–60%, depending on the geographical origin) is still unknown (Durr, 2010). The SCAs 1–3, 6–7, 17 and dentatorubral-pallidoluysian atrophy (DRPLA) are due to the expansion of CAG triplets coding for polyglutamine. SCA10, SCA12 and SCA31 are due to the expansion of non-coding regions of genes. The remaining SCAs are due to conventional mutations. The mechanisms of the SCAs affecting the PF-PC synapse are summarized in Figure 3.

#### SCA1

In several types of SCA the expression and/or function of mGlu_1_ receptors is altered in PCs. In SCA1, a polyglutamine repeat expansion of the protein ataxin1 causes a dysregulation in the expression of several genes, including members of the PC mGlu_1_ receptor signaling pathway (Serra et al., 2004). The protein expression levels of the mGlu_1_ receptor are reduced in the dendritic tree of PCs in SCA1 transgenic mice (Zu et al., 2004). In this model, silencing the SCA1 transgene restored both mGlu_1_ receptor expression and motor function, suggesting a causative role of this signaling pathway in ataxic symptoms (Zu et al., 2004). The reduction in the expression of mGlu_1_ receptor mRNA and protein levels in a SCA1-model mouse with severe ataxia was confirmed by Notartomaso et al. (2013). Treatment of these mice with a positive allosteric modulator of the mGlu_1_ receptor caused a long-lasting improvement of motor performance (Notartomaso et al., 2013). However, in these studies, the mGlu_1_-EPSC was not assessed, so that the role of mGlu_1_-mediated synaptic signaling at the PF-PC synapse remains uncertain. A recent report showed that, in SCA1-model mice with moderate ataxia, the mGlu_1_-EPSC is of similar amplitude as in controls, but with a prolonged time course (Power et al., 2016). In this case, the administration of a negative allosteric modulator of mGlu_1_ receptors shortened the mGlu_1_-EPSC to control levels and rescued the ataxic symptoms (Power et al., 2016). More studies are still necessary to understand the role of mGlu_1_ receptor signaling in different murine models of SCA1, in order to disclose how it affects the progression of symptoms so that an appropriate therapeutic approach can be used depending on the hyper- or hypo-activity of the mGlu_1_ receptor pathway.

#### SCA3

SCA3 is also due to polyglutamine expansion. In SCA3-model mice, there is a complete loss of the synaptically evoked suppression of excitation, which is endocannabinoid dependent (Konno et al., 2014). Synaptically evoked suppression of excitation is obtained by a brief burst of PF stimulation and requires the intradendritic Ca^2+^ signal, meditated by mGlu_1_ receptor activation, and dendrite depolarization (Maejima et al., 2001, 2005; Brown et al., 2003). For this reason, the absence of the synaptically evoked suppression of excitation is an indirect evidence that the mGlu_1_ receptor pathway is impaired in the SCA3 mouse model (Konno et al., 2014).

#### SCA5

SCA5 is due to mutations of beta-III spectrin, which is a scaffold protein interacting with the mGlu_1_ receptor (Armbrust et al., 2014). In SCA5-model mice, the mGlu_1_ receptor is decreased in the dendritic spines of PCs, mGlu_1_-mediated Ca^2+^ responses are impaired and mGlu_1_-dependent LTP is deficient (Armbrust et al., 2014).

#### SCA27

SCA27 is due to loss-of-function mutations of the *FGF14* gene (van Swieten et al., 2003). SCA27 patients display ataxia, tremor, dyskinesia, intellectual disability and deficits in memory and executive functioning (Brusse et al., 2006). *FGF14* codes for a protein, FGF14, which belongs to the family of fibroblast growth factors (FGFs). However, in contrast to most other members of this family, FGF14 is not secreted but it is retained intracellularly and interacts with voltage-dependent sodium (Na_V_) channels to modulate their function (Goldfarb et al., 2007). Mice with a targeted deletion of *Fgf14* (*Fgf14*-KO) recapitulate the deficits of SCA27 patients (Wang et al., 2002). In this animal model, the lack of Fgf14 is responsible for deficits in neuronal excitability of cerebellar granule cells (Goldfarb et al., 2007) and PCs (Shakkottai et al., 2009). Although the impairment of action potential firing in these cells likely plays an important role in ataxic symptoms, recent reports showed that Fgf14 is necessary to preserve proper transmission at the PF-PC synapse. In fact, in granule cell-PC mixed cultures in which *Fgf14* has been deleted or knocked down, PFs show a profound suppression of neurotransmitter release (Yan et al., 2013), correlated with a deficit of voltage-gated Ca^2+^ currents in PF presynaptic varicosities (Yan et al., 2013). However, the onset of SCA27 in some patients is in the adult age, suggesting that, in the cerebellum, the PF-PC synapse is less severely affected than in culture and that other mechanisms might be involved. Indeed, in *ex vivo* cerebellar slices from *Fgf14-KO* mice, the efficacy of the PF-PC synapse is only reduced by about 40% (Tempia et al., 2015). Also in this case, the mechanism is presynaptic, so that, with the repetitive firing of PFs, synaptic facilitation is remarkably enhanced. It is likely that such synaptic facilitation compensates in great part for the deficit in neurotransmitter release by the first spike of a burst of activity. In fact, the activation of PC mGlu_1_ receptors by high frequency firing of PFs is not affected in *Fgf14*-KO mice (Tempia et al., 2015).

### Autism Spectrum Disorders

**Figure 4:**
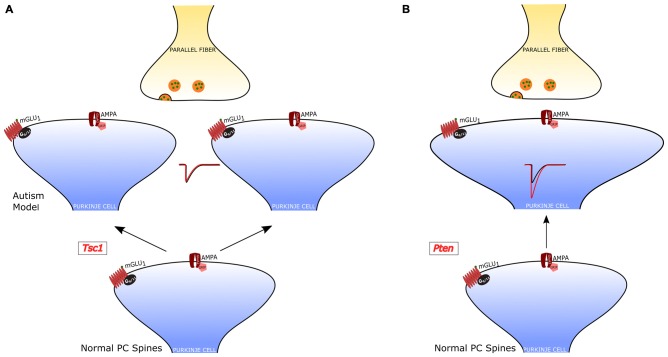
**PF-PC alterations in PC-selective animal models of autism. (A)** *Tuberous sclerosis Complex 1 (TSC1)*-knockout PCs have an increased number of dendritic spines, but normal PF-PC synaptic transmission. **(B)** *Pten*-knockout PCs have aberrant dendritic enlargements and stronger transmission at the PF-PC synapse. In a third PC-selective model of autism, the PC-*Shank2*-knockout mouse), the PF-PC synapse has not been studied, but a global *Shank2*-knockout mouse showed normal transmission at this synapse (not shown).

Regarding psychiatric disorders, the cerebellum has been strongly implied in Autism Spectrum Disorders (ASD). In fact, cerebellar malformation and PC loss are frequent findings in the analysis of autoptic material from ASD patients (Bauman and Kemper, 2005; Amaral et al., 2008). In addition, the cerebellar injury is associated with a higher incidence of ASD (Limperopoulos et al., 2007). A central role of PCs in ASD has been confirmed by recent studies in animal models. Some of these studies have even showed that a genetic modification specifically restricted to PCs is sufficient to cause symptoms related to ASD. The main findings, regarding the PF-PC synapse, of studies of PC-selective mutants are summarized in Figure 4.

The role of PCs in ASDs was first examined in a model of tuberous sclerosis, a complex disease associated with a high incidence of autism (Jeste et al., 2008). *Tuberous sclerosis Complex 1* (*TSC1*), which is one of the main genes mutated in tuberous sclerosis, was selectively knocked out in cerebellar PCs (Tsai et al., 2012). Both homozygous and heterozygous mice showed social interaction deficits and repetitive behavior, reproducing salient features of patients with ASD. Moreover, while homozygous mice also displayed ataxia, heterozygotes presented autistic trait but normal motor performance. Although heterozygous *Tsc1* mutant mice had no PC loss, the PC dendritic tree had an aberrant increase in the density of spines. It is interesting to note that in this model of ASD the efficacy of both PF-PC and CF-PC synapses was intact, but PCs showed a reduced rate of action potential firing. Thus, in this animal model, the alteration of dendritic spines was not accompanied by changes in the synaptic input impinging onto PCs, suggesting that the impairment in the output signals to the DCN is due to a deficiency in the intrinsic membrane properties regulating the generation of action potentials. This deficit and the associated behavioral symptoms could be ascribed to the loss of inhibition of mammalian target of rapamycin (mTOR) by Tsc1. In fact, an inhibitor of mTOR (rapamycin) was able to revert the autistic symptoms and the pathological alterations.

A second study examined the role of *phosphatase and tensin homolog missing on chromosome 10* (*PTEN*), a gene involved in 5–10% cases of autism (Li et al., 1997; McBride et al., 2010). PTEN negatively regulates PI3K-AKT signaling, which controls in parallel mTOR and GSK3 pathways (Song et al., 2012). A selective deletion of *Pten* in PCs was sufficient to cause autistic-like behaviors (Cupolillo et al., 2016). These *Pten* mutant mice displayed PC soma enlargement, thicker dendrites with swellings and axonal torpedoes. While at 4 months the PC number was the same in *Pten* mutant and wild-type controls, at 6 months of age the mutant PCs showed a 50% reduction in number. Behavioral and electrophysiological analyses were performed at 3–4 months of age, when PC death was not yet evident and motor performance was still normal. *Pten* mutant mice displayed deficits in social interaction and repetitive behavior, suggestive of autistic symptoms. PC action potential firing was significantly reduced, similarly to *Tsc1* mutants. However, the efficacy of the PF-PC synapse was strongly increased to double level relative to controls, through a postsynaptic mechanism. Interestingly, the CF-PC synapse was reduced via a presynaptic mechanism, as a consequence of the altered PF input or as a tentative compensation aimed at normalizing the balance of excitatory inputs to *Pten* mutant PCs.

A third study (Peter et al., 2006) focused on the postsynaptic scaffolding protein SHANK2, which is strongly linked to ASD. In accordance with ASD symptoms, mice with a PC-specific deletion of *Shank2* showed impaired sociability, and behavioral inflexibility in the form of increased perseverance in a T-maze test. In contrast to the other two PC-selective ASD models, PC-*Shank2* knockout mice had a normal PC firing frequency, but *in vivo* recordings disclosed an aberrant irregularity of PC discharge. The synaptic function was not investigated in PC-*Shank2* knockout animals, but only in mice with a global *Shank2* deletion, which showed normal PF-PC synaptic transmission but an increased frequency of postsynaptic inhibitory currents and impaired LTP of the PF-PC synapse associated with a deficit in intrinsic PC plasticity.

These three studies have in common the finding of an alteration of PC action potential firing. In *Tsc1* and *Pten* PC-selective knockout mice the frequency was reduced, while the lack of *Shank2* caused an increased irregularity of firing. These results, taken together, suggest that a preserved PC firing might be necessary to avoid ASD-related symptoms. Other features were present in some, but not in all models, like the alteration of the dendritic morphology, consisting of an increased number of spines or dendritic thickness in *Tsc1* and *Pten* mice, which in *Shank2* mice were normal. The role of the PF-PC synapse in ASD remains undefined, although the stronger efficacy in *Pten* mutants can definitely affect the afferent input signals to PCs and therefore the signal processing. Some of these alterations are likely responsible for autistic symptoms, but in order to know the relative importance of intrinsic properties relative to synaptic efficacy, it would be necessary to understand more about the mechanisms downstream of PCs. For example, optogenetic manipulations of PC firing parameters like frequency or regularity might confirm or reject the hypothesis that such signals play a central role in the pathogenesis of autism. Other relevant open questions include the consequences of an altered output from the cerebellar cortex to the DCN. Finally, we suggest that answers about this topic might arise from studies of the circuits, originating from the cerebellum, involved in the deficits of social communication, repetitive behavior and behavioral inflexibility, which are cardinal symptoms of ASD, reproduced in animal models (Lai et al., 2014).

## Conclusion

The PF-PC synapse possesses a pivotal position in the cerebellar network, being the site of maximal signal divergence and convergence. It can integrate incoming signals over different time scales, from millisecond via AMPA receptors to seconds via mGlu_1_ receptors to days up to years through plastic changes. This privileged role of the PF-PC synapse is paralleled by a variety of mechanisms of dynamic and plastic modulation. Dynamic modulation arises from different diffuse-projection neurotransmitter systems including serotonin, noradrenaline and acetylcholine. The dynamic modulation operates a fine tuning of the gain of data processing at this level. The plasticity of the PF-PC synapse can be viewed as a mechanism to store changes in the efficacy of afferent input signals to the cerebellar cortex so that the gain of each element of the PF-PC matrix can be adjusted to cope with any specific task. Such a mechanism constitutes a critical element of cerebellar motor learning and memory. The physiological importance of the PF-PC synapse explains the consequences in conditions in which it cannot work properly. In fact, alterations of this synapse are encountered in some forms of ataxia (SCA1, 3, 5, 27). The mechanisms responsible for the development of ASD are more complex. In fact, at present, it is not clear how the cerebellum is involved and how the dysregulation of the PF-PC synapse contributes to this disease. For both types of disease, ataxia and autism, the full range of physiological mechanisms has not yet been completely investigated. Furthermore, in several cases, a dysfunction of the PF-PC synapse is associated with alterations of PC firing or of other synaptic contacts. Future experiments need to clarify the specific role of each of these mechanisms in the pathogenesis of cerebellum-dependent diseases.

## Author Contributions

EH, FT, PL, MCM wrote this manuscript.

## Conflict of Interest Statement

The authors declare that the research was conducted in the absence of any commercial or financial relationships that could be construed as a potential conflict of interest.
